# Sociodemographic and Health-Risk Determinants of COVID-19 Vaccine Booster Preferences and Willingness to Pay in Singapore: Discrete Choice Experiment

**DOI:** 10.2196/87909

**Published:** 2026-07-29

**Authors:** Hooi Swang Cheng, Yi Wang, Sharon Hui Xuan Tan, Ian Yi Han Ang

**Affiliations:** 1 Saw Swee Hock School of Public Health, National University of Singapore and National University Health System, Block MD1, 12 Science Drive 2, Tahir Foundation Building, #09-01, Singapore, 117549, Singapore, 65 65164988

**Keywords:** population preferences, booster vaccination, social determinants, vaccine acceptance, vaccine hesitancy

## Abstract

**Background:**

Waning immunity and recurring COVID-19 waves point to the importance of sustained booster vaccination strategies. With repeated boosters needed to sustain population-level immunity, evaluating public preferences for COVID-19 booster vaccination is important for informing future vaccination strategies, as changing perceptions of urgency, threat, and motivation may alter willingness to receive vaccine boosters compared with initial uptake.

**Objective:**

This study aimed to explore the public’s stated preferences and implicit willingness to pay for COVID-19 vaccine boosters in Singapore and to identify the sociodemographic factors and health-risk acceptance profiles associated with these outcomes.

**Methods:**

A discrete choice experiment survey was conducted among the general population in Singapore, recruited from an online panel in June 2022. Discrete choice experiment scenarios presented hypothetical scenarios varying by booster characteristics, including effectiveness, protection duration, and cost, and risks of COVID-19 infection, hospitalization, and death. A 2-stage discrete choice design was used: participants first selected between 2 hypothetical scenarios and then reported real-life booster acceptance of their chosen option. A mixed logit model was used for analyses. We also estimated implicit willingness to pay for vaccine boosters, as well as assessed the associations of sociodemographic factors and health-risk profiles with booster acceptance or hesitancy.

**Results:**

A total of 1567 participants were included in the analysis. Overall, respondents preferred boosters that offered a longer duration of protection, particularly under higher risks of COVID-19 infection and death. Respondents were willing to pay S$1.43 for every percentage-point increase in booster effectiveness, S$4.95 (S$1=US $0.77 as of July 10, 2026) per additional month of protection, and an additional S$55.23 for a vaccine booster under the hypothetical worst-case scenario of COVID-19. Younger adults, women, individuals of non-Chinese ethnicity, those without children, people with higher socioeconomic status, individuals who had received 2 or fewer COVID-19 vaccine doses, and those with chronic conditions or on chronic disease medication were more likely to receive a booster. On the other hand, older adults, individuals with lower education or socioeconomic status, those with prior COVID-19 infection, and those exhibiting higher health-risk acceptance were less likely to accept booster vaccination.

**Conclusions:**

Our findings demonstrate how vaccine attributes, sociodemographic factors, and health-risk attitudes influence booster acceptance. These insights can guide policymakers in tailoring strategies and communication efforts that address population needs and behavioral drivers to improve booster uptake, address hesitancy, and support sustainable long-term immunization coverage, contributing to adaptive vaccination planning in the ongoing management of COVID-19.

## Introduction

Over 3 years into the COVID-19 pandemic, the World Health Organization declared the end of the Public Health Emergency of International Concern on May 5, 2023 [1]. As the pandemic transitioned into an endemic phase, many countries resumed prepandemic norms. However, the shift to endemicity does not imply that SARS-CoV-2 has become less transmissible or less virulent. Historical experiences with past influenza pandemics have shown that periodic outbreaks can occur during the endemic phase, influenced by factors such as the emergence of new viral variants, changes in population immunity, vaccine coverage, and the implementation of public health and social measures [2].

Epidemiological studies show that COVID-19 exhibits a cyclical pattern of waves during its endemic phase [2,3]. Furthermore, according to surveillance data on SARS-CoV-2 activity globally monitored by the Global Influenza Surveillance and Response System, there have been periodic peaks observed over the past few years [4]. In May 2025, several Asian regions, including Hong Kong, Thailand, Singapore, and China, reported a resurgence of COVID-19 cases [4-6]. Health authorities attributed this surge to the emergence of a highly transmissible COVID-19 variant, increased social interactions during festivities, and waning population immunity [6,7]. These developments underscore the ongoing threat posed by SARS-CoV-2 and highlight the need for sustained immunological protection.

Protective immunity through vaccination remains critical in the fight against COVID-19. However, evidence shows that vaccine-induced immunity wanes over time, potentially leading to increased susceptibility to infection and severe outcomes [8,9]. This has reinforced the importance of routine COVID-19 booster vaccinations to maintain sufficient population-level immunity and mitigate severe outcomes, particularly among vulnerable groups. Therefore, it is imperative to better understand the public’s attitudes toward COVID-19 vaccine boosters.

COVID-19 vaccines have demonstrated varying levels of effectiveness and durability against infection, hospitalization, and death [10]. Understanding the trade-offs associated with COVID-19 vaccine boosters—such as the balance between vaccine effectiveness, durability, risks associated with COVID-19, and booster out-of-pocket costs—is critical for informing adaptive and effective vaccination strategies. These insights can guide public health decisions on optimal booster intervals, target populations, and allocation of health care resources, while also accounting for opportunity cost and public acceptability. In this study, we assessed these trade-offs using a discrete choice experiment (DCE), which is a preference elicitation technique commonly used to determine the relative importance of different factors influencing people’s choices when choosing between alternatives. Several DCE studies have explored public preferences for COVID-19 vaccinations [11], but there is a lack of studies focusing on booster vaccination decisions [12,13]. Public acceptance of boosters may shift from initial vaccination uptake as perceived urgency and threat decline, pandemic fatigue sets in, and trust in vaccine safety evolves [14]. Reassessing public preferences during the transition from pandemic response to endemic management is important for anticipating future booster uptake under changing conditions. Such evidence is crucial for informing public health policies, optimizing booster schedules, and tailoring communication strategies to sustain population-level immunity.

This study aimed to explore the public’s stated preferences and implicit willingness to pay (WTP) for COVID-19 vaccine boosters in Singapore and to identify the sociodemographic factors and health-risk acceptance profiles associated with these outcomes. The results will enable differentiated outreach and health-risk communication based on various profiles of vaccine booster acceptance. They will also allow policymakers, community leaders, and researchers to identify key target groups and roll out targeted measures and interventions to increase vaccine booster uptake.

## Methods

### Study Participants

The study was a cross-sectional online survey administered through the Singapore Population Health Studies Online Panel, approved by the National University of Singapore Institutional Review Board (NUS-IRB; reference number: H-18‐011). This panel served as an online research platform that facilitated public health research on a diverse range of topics through monthly surveys related to public health. When the survey was conducted, the panel comprised 2058 active participants who were demographically representative of the general population of Singapore.

### Setting

Singapore is a city-state nation located in Southeast Asia with a population of over 5.7 million. Following the first COVID-19 case detected in January 2020, Singapore increased social distancing measures, with an eventual 2-month national partial lockdown (termed a circuit breaker) in April 2020 [15]. The COVID-19 vaccines were first introduced in Singapore in December 2020 [16]. Subsequently, a COVID-19 vaccine booster program was introduced in Singapore in September 2021 for older adults, residents of aged care facilities, and individuals with weakened immune systems, which was then gradually expanded to all other age groups [17]. In January 2022, it was announced that all individuals aged 18 and older would need to receive a vaccine booster no later than 270 days after their primary vaccination series to maintain their fully vaccinated status [18]. The survey was administered in June 2022, during the period when this policy was still in force.

As the pandemic situation stabilized, Singapore announced the easing of COVID-19 measures and the transition to an endemic phase of COVID-19 in February 2023 [19]. The nation has since been living with the endemic new normal; however, routine COVID-19 vaccination was still regarded as an important key preventive measure against severe COVID-19 illness and hospitalization [19,20].

### Survey Structure

The survey was designed with 2 main parts. The first part presented participants with two sets of DCEs, each with seven choice tasks, to elicit (1) the public’s level of acceptance of and WTP for COVID-19 vaccine boosters and (2) the nonpharmaceutical intervention measures they would be inclined to trade off if they did not want to receive and/or pay for the vaccine boosters. This manuscript focuses on the discussion of the first DCE, as the second DCE is of a different study design, with the results published elsewhere [21].

The second part of the survey consisted of 41 questions that collected sociodemographic information and assessed the level of risk acceptance and tolerance in the health domain using the Health Risk Attitude Scale (HRAS-13). The HRAS-13 is a psychometric instrument measuring risk attitudes in the health domain [22], which has demonstrated strong reliability and has been used in various population surveys and WTP studies [23,24].

### DCE Design

#### Selection of Attributes and Levels

The DCE scenarios presented a combination of different levels of the various factors of interest, focusing on vaccine booster characteristics against different risks of COVID-19 in Singapore, with the following attributes tested: (1) new cases a day: personal risk of COVID-19 infection, (2) hospitalizations a day: personal risk of hospitalization due to COVID-19, (3) deaths a day: personal risk of death from COVID-19, (4) booster effectiveness: effectiveness of the booster shot against the dominant strain, (5) effectiveness duration: duration of the booster shot maintained its level of efficacy, and (6) amount to pay for booster: the out-of-pocket booster cost.

The selection of attributes corresponding to the risks of COVID-19 was based on commonly reported statistics in news articles and press releases by the health authorities (ie, daily numbers of new COVID-19 cases, hospitalizations, and deaths), with which the respondents would be familiar [25]. The levels for the COVID-19 risk attributes were determined based on reasonable event rates relative to Singapore’s population size, supplemented by informal consultations with researchers familiar with the local epidemiological context. The attribute levels for vaccine booster’s effectiveness and effectiveness duration were selected based on what was similarly presented in the literature [26,27].

#### Experimental Design

**Figure 1.**
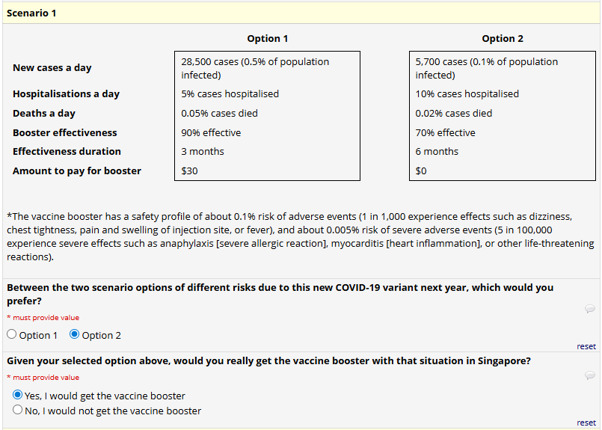
Sample of discrete choice experiment choice task.

The DCE questions were designed using Sawtooth Software Inc’s Lighthouse Studio (version 9.8) [28]. The DCE used an unlabeled choice-based conjoint design with a “dual-response none” approach. Participants were presented with a choice task consisting of 2 scenarios for them to choose in the first stage, followed by a second-stage question regarding a “no-choice option.” In the second stage, participants were asked whether they would get themselves vaccinated with a booster shot in real life given the COVID-19 situation illustrated in the option they selected in the first stage. Vaccine booster acceptance and hesitancy were defined based on participants’ responses to the second-stage question of the DCE tasks. Participants who answered “Yes, I would get the vaccine booster” were classified as demonstrating booster acceptance, while those who answered “No, I would not get the vaccine booster” were classified as demonstrating booster hesitancy. An example of a DCE choice task is presented in Figure 1.

A simulation exercise was conducted to examine the coverage matrix of the design and test the required sample size. A preliminary power analysis was conducted for the 2 DCEs. A rule of thumb has been proposed for determining the minimum sample size for aggregate-level full-profile choice-based conjoint modeling: n ≥500 *c*/*ta*, where n is the number of respondents, *t* is the number of choice tasks, *a* is the number of alternatives per task (not including the none alternative), and *c* is the largest number of levels for any attribute [29,30]. By this formula, the required number of respondents was 167. We tested the DCE designs using the Test Design function within the software. The projected standard errors for each attribute level of both DCEs were all less than 0.05.

The DCE survey underwent 2 rounds of user acceptability testing to obtain feedback on suitability, ease of understanding and phrasing, and survey length. The survey and the DCEs were refined before being rolled out to the full panel.

Each participant was randomly assigned to one of the 20 DCE questionnaire sets. Each questionnaire set had 6 unique choice tasks, totaling 120 unique choice tasks, and an additional dominant choice task for a logic check. As this was an online self-administered survey and the DCE questions can be relatively complicated compared with typical survey questions, we took a precautionary step to ensure the quality of responses by applying a dominance test, whereby one of the two choice alternatives was clearly superior. The dominance task helped to identify participants who did not understand the DCE questions or responded randomly without meaningful processing of the choice tasks. Participants who failed the dominance test were excluded from the analysis.

### Statistical Analysis

The DCE data were analyzed using a mixed logit model [31] in the following 3 steps.

#### Overall Preferences for COVID-19 Vaccine Boosters and WTP

First, a mixed logit model was used to understand participants’ overall level of acceptance of and WTP for vaccine boosters, as well as their preferences for COVID-19 vaccine boosters based on booster effectiveness and effectiveness duration. The mixed logit model considers preference heterogeneity for the attributes of the alternatives while accounting for random preference heterogeneity at the individual level.

The random utility *U_ij_* of individual *i*, for attribute *j*, where *x_ij_* is a vector of observed variables describing the attributes of the alternatives *ϵ_ij_* and is the idiosyncratic error term assumed to follow a type 1 extreme value distribution, was specified as


(1) $$U_{ij}=\beta_{i}\times x_{ij}+\epsilon_{ij}$$

where the 𝛽*_𝑖_* term is


(2) $$\beta_{i}=\beta +\eta_{i}$$

The *η_i_* term follows a multivariate normal distribution representing the random preference heterogeneity, which includes all unobserved factors that affect individuals’ preferences. The model assumes a linear combination of each utility component, including additional variables for (1) “none option,” which captures the participants’ responses in the second stage of each choice task and (2) left-and-right bias, which accounts for the tendency of participants to select only options presented on the left or the right [32].

The COVID-19 risk attributes were treated as discrete variables in the model. The price and characteristics of the vaccine booster were treated as continuous variables in the model based on the tabulation pattern of the levels of each attribute and the percentage of selections, which demonstrated a linear trend. Model selection was based on the Bayesian information criterion, comparing the model using continuous values with the model using discrete values for the 3 attributes of price, effectiveness, and protection duration of the vaccine booster, one variable at a time. The model with the minimum Bayesian information criterion value was selected.

The WTP was estimated as the ratio of the utility parameter for each attribute to the cost attribute, which was the out-of-pocket payment for a vaccine booster under the hypothetical context of this study. These estimates represent the marginal WTP for a change in a specific attribute level. For example, a WTP estimate for vaccine effectiveness reflects the additional amount respondents were willing to pay for an increase in effectiveness relative to the reference level. The WTP was presented in Singapore dollars (S$) denomination.


(3) $$WTP=-\frac{\beta_{j}}{\beta_{\text{booster price}}}$$

#### Preferences for COVID-19 Vaccine Boosters When Facing Higher Risks of COVID-19

Second, the mixed logit model included 2-way interactions between the characteristics of the vaccine booster and the risks associated with COVID-19 to examine how preferences for the vaccine booster’s characteristics varied with the risks of COVID-19 infection, hospitalization, and death. The 2 attributes, booster effectiveness and booster effectiveness duration, were interacted with the highest levels of the risks of COVID-19 attributes (ie, 57,000 new cases [1% of the population infected], 10% cases hospitalized, and 0.1% of cases died per day, respectively). The preference weight coefficients of the interaction terms indicated the changes in preference weights as the levels of booster effectiveness and booster effectiveness duration increased under each scenario of COVID-19 situation.

#### Demographic Factors Associated With COVID-19 Vaccine Booster Acceptance or Hesitancy and Characteristics of Vaccine Booster

Third, the mixed logit model was used to assess the impact of demographic factors on the level of acceptance of and WTP for vaccine boosters, and on preferences for vaccine boosters based on their characteristics. Hence, the *β_i_* term in Equation (1) was specified as


(4) $$\beta_{i}=\beta +\gamma z_{i}+\eta_{i}$$

where *𝑧_𝑖_* is the demographic variables of individual *i* that influence the mean of each attribute coefficient, and *γ* is the matrix of parameters measuring differential preferences by demographic variables. The impact of demographic factors on each attribute was considered one at a time. Only the terms with *P* values <.10 were kept in the final model. In the final model, positive coefficients indicate lower utility for booster acceptance and, therefore, greater hesitancy to accept the booster relative to the reference group, while negative coefficients indicate higher utility for booster acceptance and, therefore, greater willingness to accept the booster relative to the reference group.

Demographic factors that were considered included gender, ethnicity, age group, marital status, education level, employment status, housing type, family with children, family with children younger than 5 years, COVID-19 vaccination status, personal history of COVID-19 infection, having chronic diseases and taking medications for chronic diseases, and HRAS-13 score measuring health-related risk attitudes. Age was classified into 3 categories: younger than 40 years, 40 to 60 years, and older than 60 years. Ethnicity was classified into 2 categories: Chinese and non-Chinese (Malay, Indian, or others). Marital status was classified into 2 categories: married and not married (single, divorced, or widowed). Participants with a secondary school education or below were considered to have lower education, while participants with postsecondary school or tertiary education levels were considered to have higher education. Employment status was classified into 2 categories: full-time and part-time or not working. Information on housing type was used as a proxy for socioeconomic status (SES) [33], which was classified into 3 categories: 1- to 3-room public housing (low SES), 4- to 5-room public housing (middle SES), and private housing (high SES).

The HRAS-13 score was derived from a set of 13 questions, with participants rating their attitudes toward health risks on a 7-point Likert scale. The score was calculated by summing the 13 questions (some were reverse scored), and a higher score is indicative of having a higher level of risk acceptance in the health-related domain. The HRAS-13 score was modeled as a continuous variable.

For the analysis of booster acceptance or hesitancy, the base reference population consisted of those who were male, of Chinese ethnicity, aged 40 to 60 years, married with children but had no children under the age of 5 years, full-time workers, of middle SES, with a lower education level, had no previous COVID-19 infection, had received at least 3 shots of COVID-19 vaccine, had no chronic diseases, and had an HRAS-13 score of 0.

The level of statistical significance was set at *P*<.05. All statistical analyses were carried out using R software (version 4.2.2) [34].

### Ethical Considerations

Singapore Population Health Studies Online Panel members had previously provided informed consent as part of a master ethical approval to participate in the panel (NUS-IRB reference number: H-18‐011). The members received notification by email and/or SMS text messages about this survey on June 20, 2022, to access the survey via the provided link and were given 10 days to complete the survey on the REDCap platform. At the start of the online survey, participants were presented with a participant information sheet. Participation in the survey was voluntary and anonymous, and participants only proceeded to the survey questions after acknowledging that they had read and received a copy of the participant information sheet. Ethical approval and a waiver of documented informed consent were obtained for this study from the National University of Singapore Institutional Review Board (reference number: NUS-IRB-2020‐82). To ensure anonymity, no personal identifiers were linked to the survey responses. Participants were reimbursed S$15 (S$1=US $0.77 as of July 10, 2026) upon survey completion.

## Results

### Participant Characteristics

A total of 1651 survey responses were received from the 2058 active participants on the online research platform, corresponding to a response rate of 80.2%. Of the 1651 survey respondents, 84 were excluded; 83 respondents failed the dominance test, and 1 respondent indicated an age less than 18 years old. Among the 1567 included respondents (Table 1), the majority were female (n=914, 58.3%), of Chinese ethnicity (n=1337, 85.3%), married (n=999, 63.8%), had no chronic disease (n=1238, 79%), had attained postsecondary or tertiary education (n=1211, 77.3%), lived in 4- to 5-room public housing (n=1005, 64.1%), had children (n=917, 58.5%), and worked full-time (n=877, 56.0%). Most of them reported that they had received at least 3 doses of COVID-19 vaccine (n=1443, 92.1%) and had not contracted COVID-19 (n=940, 60%). Slightly less than half of the included respondents (n=700, 44.7%) were aged 40 to 60 years. The HRAS-13 score had a mean of 41.89 (SD 7.90); a higher score indicates higher health-risk acceptance.

Table 2 shows the appearance and selection frequencies for the different levels of the attributes used in the DCE. In about one-fifth of the choice tasks, respondents chose not to accept the vaccine booster given their options made in the first stage of the choice task.

**Table 1.** Demographic characteristics of the included respondents (N=1567).

Demographic factors	Participants
Gender, n (%)	
Female	914 (58.3)
Male	653 (41.7)
Ethnicity, n (%)	
Chinese	1337 (85.3)
Non-Chinese (Malay, Indian, and others)	230 (14.7)
Age groups (y), n (%)	
<40	499 (31.8)
40‐60	700 (44.7)
>60	368 (23.5)
Marital status, n (%)	
Married	999 (63.8)
Not married (single, divorced, separated, or widowed)	530 (33.8)
Prefer not to say	38 (2.4)
Have children, n (%)	
Yes	917 (58.5)
No	616 (39.3)
Prefer not to say	34 (2.2)
Have children younger than 5 y, n (%)	
Yes	146 (9.3)
No	1387 (88.5)
Prefer not to say	34 (2.2)
Highest level of education	
Lower education (secondary or below)	324 (20.7)
Higher education (postsecondary or tertiary)	1211 (77.3)
Prefer not to say	32 (2.0)
Employment status, n (%)	
Full-time	877 (56.0)
Part-time or not working	690 (44.0)
Housing type, n (%)	
1- to 3-room public housing (low SES^a^)	337 (21.5)
4- to 5-room public housing (middle SES)	1005 (64.1)
Private housing (high SES)	179 (11.4)
Others/prefer not to say/do not know	46 (2.9)
COVID-19 vaccination status, n (%)	
Received 2 shots and below	124 (7.9)
Received 3 shots or above	1443 (92.1)
Previous COVID-19 infection, n (%)	
Yes	619 (39.5)
No	948 (60.5)
Having a chronic disease, n (%)	
Yes	322 (20.5)
No	1245 (79.5)
On chronic medications, n (%)	
Yes	269 (17.2)
No	1298 (82.8)
Health Risk Attitude Scale-13 score, mean (SD)	41.89 (7.90)

a SES: socioeconomic status.

**Table 2.** The various attributes and their levels in the discrete choice experiment, along with the appearance and selection frequencies of the different levels for the included respondents.

Attributes and levels	Appearances, N	Selection, n (%)
Daily new cases		
5700 cases (0.1% of the population infected)	6263	3554 (56.7)
28,500 cases (0.5% of the population infected)	6189	2998 (48.4)
57,000 cases (1% of the population infected)	6352	2850 (44.9)
Daily hospitalizations		
1% of cases hospitalized	6272	3590 (57.2)
5% of cases hospitalized	6272	3100 (49.4)
10% of cases hospitalized	6260	2712 (43.3)
Daily deaths		
0.02% of cases died	6263	3543 (56.6)
0.05% of cases died	6272	3228 (51.5)
0.10% of cases died	6269	2631 (42.0)
Booster effectiveness		
50% effective	6274	1812 (28.9)
70% effective	6264	3193 (51.0)
90% effective	6266	4397 (70.2)
Booster effectiveness duration (mo)		
3	6270	2403 (38.3)
6	6287	3459 (55.0)
9	6247	3540 (56.7)
Booster price (S$)		
0	4714	2982 (63.3)
10	4694	2627 (56.0)
30	4693	2160 (46.0)
50	4703	1633 (34.7)
None option (unwilling to take vaccine booster)	9402	2045 (21.8)

### Overall Preferences for COVID-19 Vaccine Boosters and WTP

Table 3 summarizes the utility, or preference weight, from the mixed logit model. There was no evidence of left-and-right bias in choice selection (left=0.02, 95% CI –0.05 to 0.09; *P*=.48). Overall, the level of acceptance of the vaccine booster was high (coefficient for the none option=–6.58, 95% CI –6.91 to –6.24; *P*<.001), with a predicted uptake probability of 99.9% (95% CI 99.8% to 99.9%; *P<*.001). However, the random preference heterogeneity at the individual level for vaccine booster acceptance or hesitancy was large (SD for the none option=5.63, 95% CI 5.24 to 6.02; *P<*.001), indicating heterogeneous preferences among the respondents with respect to their decisions regarding accepting the vaccine booster in the real-world decision-making contexts.

The pattern of the estimated utilities for the attribute levels of risks of COVID-19 and vaccine booster characteristics was consistent with the expected utility theory [35]. Higher disutilities were exhibited for higher levels of risks of COVID-19, with increasing numbers of daily new cases, hospitalizations, and deaths. There were stronger preferences for a vaccine booster that offered stronger protection against COVID-19 in terms of its effectiveness and the duration of protection from COVID-19 variants. The relative utility increased with booster effectiveness (coefficient=0.05, 95% CI 0.04 to 0.05; *P*<.001) and booster effectiveness duration (coefficient=0.16, 95% CI 0.14 to 0.18; *P*<.001).

The WTP was generated using the coefficient of booster price as the base. The average WTP value for every percentage-point increase in booster effectiveness was S$1.43, and S$4.95 for every month increase in the duration of booster effectiveness. Respondents were willing to pay an additional S$55.23 for a vaccine booster under the hypothetical worst-case scenario of COVID-19 as presented in the DCE.

**Table 3.** Mixed logit model regression results.

Variable	Coefficient (95% CI)	*P* value	WTP^a^
Left	0.02 (−0.04 to 0.09)	.48	—^b^
None option	−6.58 (−6.91 to −6.24)	<.001	—
57,000 new cases	−0.60 (−0.69 to −0.51)	<.001	−18.59
28,500 new cases	−0.30 (−0.39 to −0.21)	<.001	−9.32
10% of cases hospitalized	−0.53 (−0.62 to −0.43)	<.001	−16.20
5% of cases hospitalized	−0.26 (−0.35 to −0.17)	<.001	−7.96
0.10% of cases died	−0.66 (−0.76 to −0.57)	<.001	−20.45
0.05% of cases died	−0.26 (−0.35 to −0.18)	<.001	−8.15
Booster effectiveness	0.05 (0.04 to 0.05)	<.001	1.43
Booster effectiveness duration	0.16 (0.14 to 0.18)	<.001	4.95
Booster price	−0.03 (−0.03 to −0.03)	<.001	—

a WTP: willingness to pay.

b Not applicable.

### Preferences for COVID-19 Vaccine Booster When Facing Higher Risks of COVID-19

Results shown in Table 4 indicate that under the scenarios with the highest numbers of daily new cases and daily deaths, individuals’ utility for booster effectiveness duration further increased (coefficient=0.06, *P*<.005, respectively). However, people became more price sensitive in the scenario with the highest number of daily deaths (coefficient=−0.01, 95% CI −0.01 to −0.00; *P*=.04).

**Table 4.** Mixed logit model with 2-way interactions between characteristics of vaccine booster and the highest levels of COVID-19 risk as illustrated in the discrete choice experiment.

Variable	Coefficient	*P* value
Left	0.04 (−0.03 to 0.11)	.25
None option	−6.55 (−6.91 to −6.20)	<.001
57,000 new cases	−0.77 (−1.32 to −0.22)	.006
28,500 new cases	−0.35 (−0.45 to −0.26)	<.001
10% of cases hospitalized	−0.23 (−0.77 to 0.32)	.42
5% of cases hospitalized	−0.27 (−0.36 to −0.17)	<.001
0.10% of cases died	−1.06 (−1.58 to −0.54)	<.001
0.05% of cases died	−0.30 (−0.39 to −0.20)	<.001
Booster effectiveness	0.05 (0.04 to 0.05)	<.001
Booster effectiveness duration	0.13 (0.10 to 0.15)	<.001
Booster price	−0.03 (−0.04 to −0.03)	<.001
57,000 new cases × booster effectiveness	−0.00 (−0.01 to 0.00)	.28
57,000 new cases × booster effectiveness duration	0.06 (0.02 to 0.10)	.004
57,000 new cases × booster price	0.00 (−0.00 to 0.01)	.47
10% of cases hospitalized × booster effectiveness	−0.00 (−0.01 to 0.00)	.23
10% of cases hospitalized × booster effectiveness duration	−0.01 (−0.05 to 0.04)	.75
10% of cases hospitalized × booster price	0.00 (−0.00 to 0.01)	.79
0.10% of cases died × booster effectiveness	0.00 (−0.00 to 0.01)	.48
0.10% of cases died × booster effectiveness duration	0.06 (0.02 to 0.09)	.003
0.10% of cases died × booster price	−0.01 (−0.01 to −0.00)	.04

### Demographic Factors Associated With COVID-19 Vaccine Booster Acceptance or Hesitancy and Characteristics of Vaccine Booster

**Figure 2.**
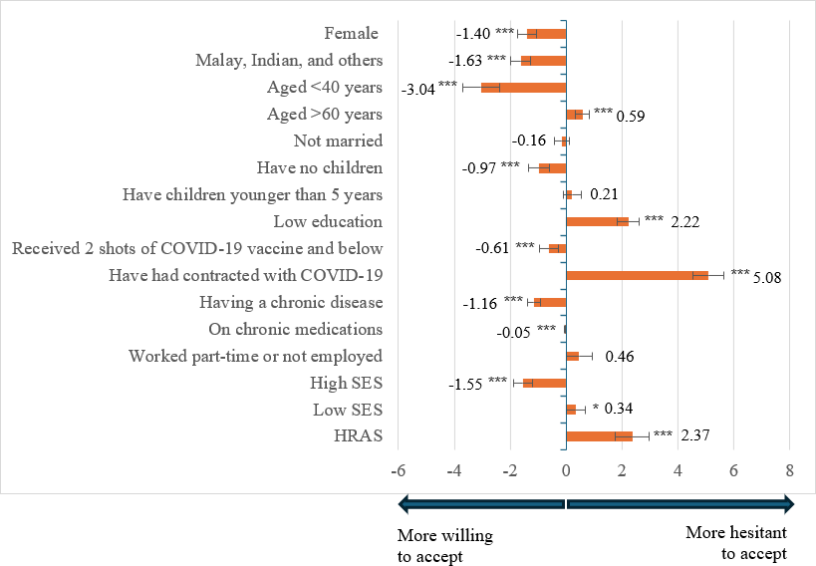
Association between demographic factors and COVID-19 vaccine booster acceptance or hesitancy. HRAS: health-risk attitude scale score; SES: socioeconomic status. ***P<.001*;* *P<.05*.*

Of the 1567 included respondents, 1469 provided complete demographic information and were included in this analysis. Ninety-eight respondents were excluded due to “Prefer not to say” responses. Demographic factors associated with vaccine booster acceptance were female, non-Chinese ethnicity, aged younger than 40 years, having no children, high SES (defined as living in private housing), having received 2 shots of COVID-19 vaccine or less, having a chronic disease, and on chronic disease medication. On the other hand, vaccine booster hesitancy was noticed among those aged older than 60 years, with a lower education level, low SES (defined as living in 1- to 3-room public housing), who had previously contracted COVID-19, and with higher health-risk acceptance (see Figure 2). However, there were no strong associations observed between demographics and characteristics of vaccine booster. Results are provided in Multimedia Appendix 1.

## Discussion

### Principal Results

This study examined the associations between sociodemographic characteristics, individual health-risk profiles, and public preferences for COVID-19 vaccine boosters, including out-of-pocket cost, among the Singapore population using a DCE. Overall, our study showed that booster preferences varied across sociodemographic and health-risk subgroups. Individuals with lower educational attainment (coefficient=2.22, 95% CI 1.83-2.63), older adults (coefficient=0.59, 95% CI 0.34-0.85), and those living in smaller public housing types (coefficient=0.34, 95% CI 0.01-0.69) showed lower acceptance of vaccine boosters, underscoring persistent socioeconomic gradients in booster uptake. Health-risk profiles also played an important role; we found that individuals with higher health-risk acceptance were less likely to accept booster vaccination (coefficient=2.37, 95% CI 1.77-2.97). These findings provide important insights into the key drivers of booster vaccination decisions and highlight critical subgroups that may require tailored communication and policy interventions to sustain booster uptake.

### Comparisons With Prior Work

Respondents were willing to pay more to gain the benefit, that is, an additional S$4.95 for every month increase in the duration of vaccine booster effectiveness. These findings imply that the risks of COVID-19 may influence people’s WTP for vaccines. Respondents were willing to pay a much higher amount (ie, an additional S$55.23) for vaccine boosters to avoid the hypothetical worst-case scenario of COVID-19. The estimated average WTP value for every unit percent increase in booster effectiveness was S$1.43, which was much lower than that reported in the Australian study for COVID-19 vaccine (ie, Aus $23.92) [36]. This finding was not unexpected, as people are less willing to pay for the vaccine out of pocket considering the recurring costs of repeated vaccine boosters required. Additionally, Singapore has provided and continues to provide the COVID-19 vaccines and the booster doses free of charge.

In the multivariable analysis, we found that the health-risk profile and several sociodemographic characteristics, including age, gender, ethnicity, education level, SES, having children, chronic conditions, COVID-19 vaccination status, and previous history of COVID-19 infection, were associated with vaccine booster acceptance or hesitancy. Our findings identified that individuals with higher health-risk acceptance were less likely to accept a vaccine booster and preferred a vaccine booster with lower effectiveness. Moreover, our findings of greater booster acceptance among those who did not complete their primary vaccination series, had a chronic disease, and were taking medication for a chronic disease also provide evidence that individuals with higher risks of COVID-19 infection had a higher utility for vaccine boosters.

We found that individuals who reported that they had contracted COVID-19 were more hesitant to accept the vaccine booster. A study showed that natural immunity provides a comparable level of protection against new COVID-19 infection as vaccine-induced immunity [37]. Those with a previous history of COVID-19 infection might feel confident in the protection by infection-induced immunity and/or not be too concerned about the effects of an infection. As such, they might deem an additional booster dose as redundant [38]. Interestingly, we found that individuals with no children were more likely to accept boosters, suggesting that individuals with and without young dependents may perceive the risks and benefits of vaccine boosters differently for adults and children. As of April 2023, the Singapore Ministry of Health reported that more than two-thirds of children aged 5 to 11 years had not completed the vaccine doses required for minimum protection against COVID-19 [39]. Despite having confidence and trust in the COVID-19 vaccine for adults, parents, who are often the decision makers of child vaccination, may have concerns about the safety of the COVID-19 vaccine for their children and may reject booster doses for themselves when they are hesitant to vaccinate their children [40,41].

Our findings corroborate previous literature showing greater booster hesitancy among those with lower educational attainment [41-43] and greater booster acceptance among those with high SES groups [42,44] and those with a chronic disease [45]. On the other hand, we found some contrary results compared to previous studies. Our findings identified that there was greater booster acceptance among females, while the opposite was previously found in a meta-analysis [42]. A previous study conducted in Singapore found no gender differences in COVID-19 vaccine hesitancy [46]. However, greater vaccine acceptance was observed among females in a population-based study conducted in Hong Kong [47].

Although most studies have reported lower COVID-19 booster hesitancy among older adults [41,42,48], this study found that booster hesitancy was higher among older adults, whereas younger individuals exhibited greater acceptance of COVID-19 booster doses in the multivariable-adjusted model. Our findings are consistent with a recent systematic review and a study from Malaysia that found higher levels of booster hesitancy among older adults, suggesting that age-related differences in booster acceptance may vary across populations and contexts [43,49]. Furthermore, recent national data from Singapore indicated that, as of June 2024, booster vaccination rates among older adults aged 60 years and older remained low, lending further support to our findings [50]. Greater effort should be put forth on educational campaigns and in targeting public health messaging to individuals of older age, lower educational attainment, and lower SES to improve vaccine booster uptake among these subpopulations. This study also found that individuals of non-Chinese ethnicity were more likely to accept vaccine boosters, suggesting that the efforts made by the government and Muslim religious authorities to reduce vaccination barriers among minority groups have been effective in addressing earlier hesitancy [46].

The predicted booster uptake probability observed in this study was high, nearly 100%. Respondents may have been influenced by the prevailing policy environment, specifically the booster vaccination recommendations and the requirement to maintain fully vaccinated status, both of which were introduced in early 2022 and remained in effect at the time of data collection. This may partly reflect the broader policy environment, as well as social desirability bias in responses, whereby respondents reported choices that aligned with perceived public health expectations rather than reflecting their actual future behavior. It may also be attributable to the characteristics of the study sample, which was highly vaccinated: 92% of respondents self-reported having received three or more vaccine doses at the time of the survey. This suggests that respondents were already predisposed toward vaccine acceptance, and the near-universal predicted uptake may therefore reflect a pre-existing provaccination orientation and prior vaccination behavior, rather than the effects of the DCE attributes alone.

As with all DCE studies, our estimates reflect stated rather than revealed preferences. A positive attitude toward a vaccine booster does not necessarily always translate to actual uptake in reality [45]. The near-universal predicted uptake observed in this study contrasts sharply with actual booster uptake in Singapore, where about half among seniors aged 60 years and older had received an additional booster dose by November 2023 [51], and fewer than 20% of the local population had received a COVID-19 booster dose within the preceding year by May 2024 [52]. This discrepancy may also reflect the influential role of policy mandates and perceived disease risk in shaping vaccination behavior. During the initial booster rollout, uptake was high, with approximately 84.5% of eligible individuals having received a first booster dose by January 2022 [53], likely reflecting the combined effects of vaccination-differentiated measures and heightened concern about COVID-19. When these measures were withdrawn and the perceived threat of COVID-19 declined, uptake of subsequent annual boosters fell markedly.

On the other hand, the discrepancy between stated preferences and actual booster uptake may partly reflect factors beyond vaccine attributes and sociodemographic characteristics. Previous studies have highlighted the influence of psychological determinants, including safety, vaccine confidence or side effects, and trust, which may affect whether favorable intentions ultimately translate into vaccination behavior [54,55].

### Limitations

This study has several limitations. First, as this was a cross-sectional study, it captured a snapshot of public sentiment at a single point in time and could not account for potential changes in attitudes over time. As most countries have now transitioned to the endemic phase of COVID-19, public attitudes toward vaccine boosters are likely to evolve. Given that the study was conducted before the formal transition to the endemic phase, the findings may not be fully generalizable to the current endemic context. However, this timing allowed the study to capture public preferences during a critical phase when booster policy was being adapted from pandemic response to longer-term management under evolving risk perceptions and policy signals. Future research employing longitudinal designs would be valuable for monitoring shifts in public perceptions and acceptance of boosters in response to changing epidemiological conditions or policy cycles.

One other limitation is that DCEs may be subject to hypothetical bias. Nevertheless, we sought to reduce hypothetical bias by emphasizing the application to real-life choices. Future studies can assess the concordance between stated and revealed preferences within individuals [56], and expand on cheap-talk scripts to further reduce hypothetical bias [57]. Another limitation is that the worst-case scenarios included in the DCE (57,000 daily new cases, 10% of cases hospitalized, and 0.10% of cases died) exceeded the reported daily case burden observed in Singapore and may have been perceived as less realistic by some respondents. However, these levels were designed to represent a severe outbreak scenario and to capture preferences across a broad range of epidemiological conditions. Nevertheless, respondents who failed the dominant choice task were excluded from the analysis, providing some reassurance that participants remained engaged with and were able to meaningfully evaluate the hypothetical scenarios.

Finally, although the participant pool was demographically representative of the Singaporean population, the study was limited to those who were able to read and understand English. About 20% of the resident population aged 15 years and older are illiterate in English, with over half of them being older adults aged 55 years and older [58]. Future research could include other languages to capture individuals not included in the current study and improve the generalizability of the findings. This could be especially important to further inform comprehensive policy decision-making aimed at more targeted public health messaging. Despite these limitations, the study offers valuable insights into the determinants of booster acceptance and provides evidence to inform vaccination planning as COVID-19 management continues to evolve.

### Conclusions

Vaccine boosters are a vital tool to maintain societal resilience against COVID-19. Our findings demonstrated that individuals generally preferred boosters offering longer-lasting protection, particularly in the context of heightened COVID-19 risks of infection and death. These findings underscore the dynamic nature of public attitudes, which may shift in response to evolving policy measures, vaccination recommendations, and epidemiological trends. In addition, this study identified the factors associated with booster acceptance and hesitancy, providing valuable insights that can shape targeted public health strategies and messaging to promote the uptake of vaccine boosters. These findings may extend beyond COVID-19, offering guidance for the development of vaccination policies for future pandemics and epidemics. The estimates of the WTP values in this study may also guide pricing decisions for COVID-19 vaccination in the long term. As preferences for preventive health interventions are highly context dependent, the results should be interpreted in the context of the period in which the study was conducted and may not fully represent the current setting or true endemic-phase perceptions.

## Supplementary material

10.2196/87909 Multimedia Appendix 1 Associations between demographic factors and COVID-19 vaccine booster acceptance/hesitancy and characteristics of vaccine booster using mixed logit model.

## ABBREVIATIONS

DCE: discrete choice experiment
HRAS-13: Health Risk Attitude Scale-13
NUS-IRB: National University of Singapore Institutional Review Board
SES: socioeconomic status
WTP: willingness to pay
