# Supplementary material for: Sociodemographic and Health-Risk Determinants of COVID-19 Vaccine Booster Preferences and Willingness to Pay in Singapore: Discrete Choice Experiment
Source: Interact J Med Res. 2026 Jul 29;15:e87909. doi: 10.2196/87909 (PMC13418094; doi:10.2196/87909)
Supplement: Multimedia Appendix 1 [file ijmr-v15-e87909-s001.docx]

## Supplementary Table

Supplementary Table. Associations between demographic factors and COVID-19 vaccine booster acceptance/hesitancy using mixed logit model

|  | Coefficient | 95% CI | P value |
| --- | --- | --- | --- |
| Association between demographic factors and COVID-19 vaccine booster acceptance/hesitancy | | | |
| *Associated with None option* | | | |
| Female | -1.40 | (-1.74, -1.07) | *<.001* |
| Malay, Indian, and others | -1.63 | (-1.99, -1.28) | *<.001* |
| Aged <40 years | -3.04 | (-3.70, -2.39) | *<.001* |
| Aged >60 years | 0.59 | (0.34, 0.85) | *<.001* |
| Not married | -0.16 | (-0.44, 0.12) | *.25* |
| Have no children | -0.97 | (-1.37, -0.59) | *<.001* |
| Have children younger than 5 years | 0.21 | (-0.11, 0.53) | *.18* |
| Lower education | 2.22 | (1.83, 2.63) | *<.001* |
| Worked part-time or not employed | 0.46 | (-0.01, 0.93) | *.05* |
| High SES | -1.55 | (-1.89, -1.23) | *<.001* |
| Low SES | 0.34 | (0.01, 0.69) | *.05* |
| Received 2 shots of COVID-19 vaccine and below | -0.61 | (-0.96, -0.27) | *<.001* |
| Have had contracted with COVID-19 | 5.08 | (4.54, 5.63) | *<.001* |
| Having a chronic disease | -1.16 | (-1.40, -0.93) | *<.001* |
| On chronic medications | -0.05 | (-0.07, -0.04) | *<.001* |
| HRAS-13 score* | 2.37 | (1.77, 2.97) | *<.001* |

*HRAS-13 score is treated as continuous variable.
